# Harnessing the wandering mind: The role of perceptual load

**DOI:** 10.1016/j.cognition.2009.02.006

**Published:** 2009-06

**Authors:** Sophie Forster, Nilli Lavie

**Affiliations:** Institute of Cognitive Neuroscience and Research Department of Cognitive, Perceptual and Brain Sciences, University College London, Gower Street, London WC1E 6BT, UK

**Keywords:** Task-unrelated thoughts, Mind-wandering, Perceptual load, Attention, Response competition, Distractor interference

## Abstract

Perceptual load is a key determinant of distraction by task-irrelevant stimuli (e.g., Lavie, N. (2005). Distracted and confused?: Selective attention under load. *Trends in Cognitive Sciences, 9*, 75–82). Here we establish the role of perceptual load in determining an internal form of distraction by task-unrelated thoughts (TUTs or “mind-wandering”).

Four experiments demonstrated reduced frequency of TUTs with high compared to low perceptual load in a visual-search task. Alternative accounts in terms of increased demands on responses, verbal working memory or motivation were ruled out and clear effects of load were found for unintentional TUTs. Individual differences in load effects on internal (TUTs) and external (response-competition) distractors were correlated. These results suggest that exhausting attentional capacity in task-relevant processing under high perceptual load can reduce processing of task-irrelevant information from external and internal sources alike.

## 1 Introduction

A main goal of attention research is to understand the determinants of successful focused attention that allows for minimal distraction by goal-irrelevant information. This fundamental issue has stimulated much research over the past four decades (e.g., see Kahneman and Treisman (1984), Lavie and Tsal (1994), Lavie (1995) for reviews) and a major determinant of focused attention that has been highlighted is the level of perceptual load in a task. The role of perceptual load in attention has been elucidated within the perceptual load theory (Lavie, 1995; Lavie & Tsal, 1994), which suggests that distractor processing critically depends on the availability of attentional capacity and can thus be prevented when the relevant-task processing involves sufficient high perceptual load to engage full attentional capacity.

Evidence in support of this claim has been found in many studies demonstrating that distractor processing is significantly reduced with tasks of high (compared to low) perceptual load (e.g., Lavie, 1995; Rees, Frith, & Lavie, 1997; see Lavie, 2005, for review). This conclusion has generalized across various manipulations of perceptual load (that have either involved a greater number of items requiring identification in the high load conditions or a greater complexity of the perceptual task, see Lavie, 2005, for review) and across various measures of distractor processing. For example, perceptual load has been shown to reduce (and indeed typically eliminate) distractor interference effects measured with response competition (Lavie, 1995; Lavie & Cox, 1997; Lavie, Ro, & Russell, 2003) and negative priming (Lavie & Fox, 2000). Perceptual load has also been shown to modulate brain activity related to distractors whether these are visible (Rees et al., 1997; Schwartz et al., 2005; Yi, Woodman, Widders, Marois, & Chun, 2004) or even invisible (Bahrami, Lavie, and Rees, 2007).

Recent studies have demonstrated that perceptual load effects can overcome individual differences in distractibility (Forster & Lavie, 2007) and can also eliminate the effects of highly salient distractors that, like many daily life distractors, cause interference despite bearing no relationship to the task currently being performed (Forster & Lavie, 2008a; Forster & Lavie, 2008b). However, in daily life sources of distraction may not only be found in the external environment, but also in the form of internally generated distractions such as task-unrelated thoughts (TUTs). For example, a person may be distracted from reading this article by the intrusion of a thought about an unrelated issue - perhaps some salient recent event in his or her daily life. The purpose of this paper is to clarify whether the established role of perceptual load in determining task-irrelevant processing would also apply to internal sources of potential distraction, such as distraction by TUTs.

We reasoned that, similarly to the role of load in processing task-irrelevant information from external sources, the processing of task-irrelevant information from internal sources such as mind-wandering may also be determined by perceptual load. The load research we previously mention has established that processing of task-irrelevant stimuli with very different contents (e.g., verbal vs. visual and complex stimuli such as words or visual scenes vs. simple stimuli such as letters or contrast and motion, Brand-D’Abrescia & Lavie, 2007; Lavie, 2006; Macdonald & Lavie, 2008; Rees et al., 1997; Schwartz et al., 2005) and sources (e.g., different sensory modalities, conscious as well as unconscious processes, e.g., (Bahrami, Carmel, Walsh, Rees, & Lavie, 2008; Cartwright-Finch & Lavie, 2006; Dalton, Lavie, and Spence, 2008; Macdonald & Lavie, 2008) is modulated by the level of load in the task-relevant processing. These findings provide support for the Load Theory claim that the processing of any task-irrelevant information (be it verbal or visual, simple or complex, conscious or unconscious, in one sensory modality or another, and so forth) requires limited-capacity attention and can therefore occur only when the processing of task-relevant information leaves some spare attentional capacity (under conditions of low perceptual load). We thus reasoned that higher perceptual load that would engage more attentional capacity in the processing of task-relevant information may reduce the processing of task-irrelevant information not only from external sources (e.g., visual distractors) but also from internal sources (e.g., mind-wandering) in the present study.

### 1.1 Mind-wandering measures and previous findings

In comparison to the extensive body of research examining the attention principles that govern distraction by external stimuli, the topic of attention and distraction by mind-wandering has been relatively understudied, perhaps due to lack of any truly objective method for directly measuring the occurrence of such a highly subjective phenomenon. However, laboratory measures of mind-wandering have been developed (e.g., measuring TUT occurrence with probe-caught methods, whereby on the appearance of a probe the subject has to report whether or not they had just experienced TUT), and studies using these methods suggest that mind-wandering is a ubiquitous and potent source of distraction: TUTs often occur unintentionally and interfere with performance on a range of tasks from signal detection tasks to encoding and reading tasks (Schooler, Reichle, & Halpern, 2004; Segal & Fusella, 1970; Smallwood, Baracaia, Lowe, & Obonsawin, 2003; Smallwood, O’Connor, Sudberry, Haskell, & Ballantyne, 2004; Smallwood, McSpadden, & Schooler, 2007; see also Smallwood & Schooler, 2006; Smallwood, Fishman, & Schooler, 2007 for review).

Despite these apparent distracting effects of mind-wandering, however, no study as yet has demonstrated any causal role for focused attention in determining levels of TUT. A number of studies have directly manipulated task factors that were found to decrease the level of task-unrelated thoughts,^1^ but none has yet used an established manipulation of focused attention. Moreover, in almost all cases these manipulations are likely to have interfered directly with the very ability to produce or maintain a thought. Thus the role of focused attention in TUTs (or mind-wandering) remains unclear.

For example, it has been shown that working memory load can reduce TUT (Teasdale et al, 1995; Teasdale, Proctor, Lloyd, & Baddeley, 1993), yet working memory is clearly needed in order to provide a mental work space for thought (for example one would need to maintain the start of the thought in order to develop its semantics in a coherent manner in which the end relates to the start, see Baddeley (1986)). It has also been shown that performing a task relative to no task at all or increasing stimulus presentation rates can reduce the rates of TUT reports (e.g., Giambra, 1995; McKiernan, D’Angelo, Kaufman, & Binder, 2006). However, not only do conditions of task performance (compared to no task performance) and faster (vs. slower) presentation rates involve a higher working memory load, but they also involve increased demands on responses (as with higher presentation rate the response rate is also higher). The simple act of making a response has been shown to directly interfere with the rate of TUT reports (e.g., see Antrobus (1968)). By contrast, here we ask whether it is possible to reduce task-unrelated thoughts by engaging more attentional capacity in a task with high (compared to low) perceptual load, without directly drawing on thought or response components that are clearly part and parcel of the production or report of any thought.

The suggestion of a recent review of the mind-wandering literature that the rate of TUTs reported is higher in tasks that require only “superficial engagement” compared to those that require moderate or deeper levels of engagement (Smallwood, Fishman et al., 2007) is encouraging for our present load hypothesis. However, the broad term “task-engagement” may encompass a number of factors in addition to attention (e.g., changes in the overall level of motivation, interest and arousal, as well as the engagement of processes such as working memory and thought) and the tasks presumed in the review to involve differing levels of task engagement (e.g., signal detection tasks were assumed to involve superficial engagement, whereas reading tasks were assumed to always involve deep engagement) also differed in terms of many of these factors. Therefore any variation in the rate of TUTs reported during these different tasks cannot be safely attributed solely to the level of attentional engagement, rather than to these other factors.

Recent findings of an association between the speeding of responses in a go/no go task (which may reflect less careful task-performance stemming from reduced task-engagement) and a higher rate of TUT reports (Smallwood et al., 2004; Smallwood, McSpadden et al., 2007) also provide some suggestive evidence that the level of attentional engagement in a task may determine TUT. However, changes in the speed of responses may be driven by a number of factors other than attentional engagement in the task (e.g., practice, arousal, motivation). Furthermore, in the absence of any manipulation of attention it is impossible to determine whether it was the speeding of responses (and putative task-disengagement it reflects) that led to a higher rate of TUT or whether the occurrence of TUT led to the putative attention disengagement and hence the speeding of the task responses.

The present study is thus the first to examine whether focused attention plays a causal role in determining mind-wandering, by directly manipulating the level of demand on attentional capacity (through our manipulation of perceptual load) in visual search: a paradigmatic and widely used attention task.

Finally, it is important to note that some effects of a secondary task or working memory load on mind-wandering have previously been interpreted as indicating that mind-wandering draws on central executive resources (e.g., Smallwood & Schooler, 2006; Teasdale et al., 1995) and in some cases these have been referred to as “executive attention” (Smallwood & Schooler, 2006). However recent advances in attention research demonstrate that ‘attention’ cannot be used as an umbrella term encompassing all limited-capacity processes, instead it is important to distinguish between the effects on focused attention of capacity limits in perception vs. in executive control processes. Whereas exhausting perceptual capacity in tasks of high perceptual load reduces distractor processing and thus improves focused attention, load on executive control processes such as working memory during selective attention tasks has the opposite effect of increasing rather than decreasing distractor processing. Since load on working memory reduces its availability to actively maintain focused attention in line with current stimulus processing priorities the irrelevant distractor stimuli gain more processing access (e.g., De Fockert, Rees, Frith, & Lavie, 2001; Lavie, 2000; Lavie, Hirst, de Fockert, & Viding, 2004).

We consider the implications of this dissociation for mind-wandering later (in the General Discussion). For now we note that in the present study, given our focus on perceptual load rather than executive control, our manipulation of perceptual load was designed to minimize any confounding involvement of higher levels of executive control resources with the increase in perceptual load.

To this end we incorporated thought probes into a typical perceptual load paradigm involving a visual search task in which the number of search non-targets was varied (from one to six items) to produce high or low perceptual load (e.g., Lavie, 1995; Lavie, 2005; Lavie & Cox, 1997). The manipulation of perceptual load through the number of search non-targets^2^ (often termed “set size”) is very well established (see Lavie (2005) for review) and has been shown to reduce distractor perception and related activity in the visual cortex in many previous studies (see Macdonald and Lavie (2008) for a recent demonstration and review).

Notice in particular that the controlled aspects of the task (e.g., maintaining the target template in working memory) remain constant across the levels of perceptual load, only the number of search items is varied. As such, this increase in the number of items places demands on perceptual search processes (e.g., serial spatial scanning of a greater number of search items, see Treisman (1988), Treisman and Gelade (1980)) rather than control processes. Indeed, previous research has indicated that increasing perceptual load in this task does not interfere with working memory task performance, nor does it interact with the effects of working memory load on distraction (e.g., Lavie et al., 2004; Logan, 1978). Thus, any effects of perceptual load on TUT in this task are unlikely to reflect direct interference with thought components, or with their executive control (e.g., by blocking working memory).

## 2 Experiment 1

In Experiments 1a and 1b participants performed a letter search task with high and low perceptual load, during which they responded to thought probes. In the original perceptual load paradigm on which our task is based (Lavie & Cox, 1997) feedback was given for errors in the form of a brief “beep” tone. As the error rate is typically higher in the high load condition this means that the high load condition would be associated with more frequent feedback tones. It was therefore important to establish that any reduction in the level of TUT reports under high perceptual load did not simply reflect an effect of the greater number of feedback tones in this condition. For example, as abrupt onsets have been shown to capture attention during the performance of an external task (e.g., see Jonides and Yantis (1988), Yantis and Jonides (1984)), the greater number of abrupt sound onsets in the high load condition might capture attention towards the external environment and task and therefore away from any task-unrelated thoughts. In addition, the higher level of feedback may have increased motivation to remain on task in the high (compared to low) load condition. Indeed feedback tones can be used to improve participants’ ability to maintain sustained attention to a task (e.g., Manly et al., 2004). For this reason, we ran two versions of Experiment 1, with and without the error feedback. Participants in Experiment 1a were presented with error feedback, but those in Experiment 1b were given feedback only during the practice trials and not during the main task performance.

### 2.1 Method

#### 2.1.1 Participants

All participants in the reported experiments were volunteers recruited from University College London subject pool and had normal or corrected to normal vision. Ten participants (three males, 20–35 years old) participated in Experiment 1a. Fourteen participants (three males, 8–35 years old) participated in Experiment 1b. The data from one additional participant in Experiment 1b was not included in the analysis as this participant performed at chance level on the task.

#### 2.1.2 Stimuli and procedure

E-Prime was used to program and run the experiment. All stimuli were presented in white on a black background, on a 15 in. monitor and at a viewing distance of approximately 60 cm. Each trial in the visual search task began with 500 ms black screen interval, followed by a fixation point presented for 500 ms in the centre of the screen. This was followed immediately by the stimulus display, consisting of six letters arranged to form a circle (with the radius subtending 1.6° degrees of visual angle), presented for 100 ms. Participants were required to search the display for a target letter (either *X* or *N*, subtending 0.6° × 0.4°) and respond using the numerical keypad by pressing the 0 key if the target was an *X* and the 2 key if the target was an *N*. In Experiment 1a, a 90 ms beep sounded on incorrect responses or if the participant failed to respond within a 3000 ms time window. In Experiment 1b, participants were given this same feedback during the practice trials, but were given no feedback during the main experimental trials. In the low load condition the five non-target letters were all small Os (subtending 0.15°), whereas in the high load the non-target letters were heterogeneous angular letters (selected at random from H, K, M, Z, W, V). All combinations of target identity and position were fully counterbalanced.

Participants performed 3 slower low and high load example trials before receiving definitions and examples of task-related and task-unrelated thoughts. Task related thoughts were defined as being thoughts about any aspect of the task being performed at that moment, with examples being “where is the target letter?” or “oops, I’ve pressed the wrong button”. Task-unrelated thoughts were defined as being thoughts about anything other than the task being performed, with examples being “my lecture this morning was boring.” or “I must stop by the supermarket on the way home.” Participants then completed twelve practice trials of each level of load with a practice thought probe after each set of twelve trials. The thought probe consisted of the question “What were you thinking just now?”. Participants were instructed to report the thought that had been passing through their mind in the moment just before the probe appeared. Onscreen instructions asked the participant to press A if they were thinking about the task that they were performing, and to press Z if they were thinking about something unrelated to the immediate task. Participants were told that during the letter task they should keep their fingers ready on the relevant keys and try to focus on the task in order to respond as fast as possible while also maintaining a high level of accuracy. They were told that there would be no need to prepare for the thought probes (e.g., by keeping their fingers on the response keys) before they appear on screen, as instructions for the responses to the thought probes would be presented with each probe. They were also reassured that there was no right or wrong answer to the probe question and that their probe responses were not being timed, so they should therefore simply respond honestly to the question. Participants then completed eight high load and eight low load blocks of 48 trials in the order ABBAABBAABBAABBA (counterbalanced between participants), with a thought probe presented at the end of each block.

### 2.2 Results and discussion

All RT analyses in the experiments reported here were conducted only on trials to which a correct response was made. Analyses of RTs and percentage error (PE) rates in the high and low load conditions replicated previous findings (e.g., Lavie & Cox, 1997), confirming that the increase in the relevant search set size (through the number of angular letters) was effective in increasing load. Participants’ RTs were significantly longer under high load (for Experiment 1a *M* = 644 ms; for Experiment 1b *M* = 706 ms) than under low load (for Experiment 1a *M* = 486 ms; for Experiment 1b *M* = 490 ms, *t* (9) = 14.11, SEM = 11.20, *p* < .001, *d* = 9.41 for the difference in Experiment 1a; *t* (13) = 11, SEM = 19.66, *p* < .001, *d* = 6.10 for the difference in Experiment 1b).

The high load condition was also associated with a significantly higher PE rate (for Experiment 1a, *M* = 16.4%; for Experiment 1b, *M* = 9.9%) than the low load condition (for Experiment 1a, *M* = 7.2%, for Experiment 1b, *M* = 4.7%), *t* (9) = 5.18, SEM = 1.78, *p* = .001, *d* = 3.45 for the difference in Experiment 1a; *t* (13) = 4.99, SEM = 1.03, *p* < .001, *d* = 2.77 for the difference in Experiment 1b.

**Fig. 1:**
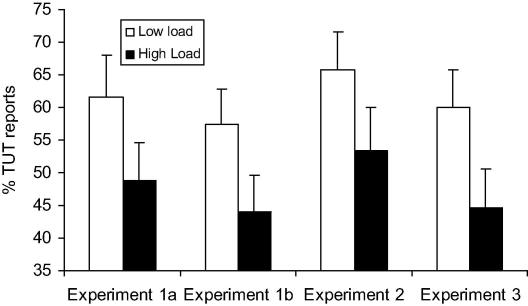
Percentage of probes at which a task-unrelated thought (TUT) was reported under high and low perceptual load in Experiments 1–3.

Across the load conditions, the percentage of thought probes to which a TUT was reported varied between 25% and 88% (*M* = 52.63%, S.D. = 16.19). Importantly, paired *t*-tests confirmed our main prediction that perceptual load would reduce TUTs: As can be seen in Fig. 1, the percentage of TUTs reported was significantly reduced in the high load condition (for Experiment 1a, *M* = 48.9%, for Experiment 1b, *M* = 44%), compared to the low load condition (for Experiment 1a, *M* = 61.6%, for Experiment 1b, *M* = 57.5%), *t* (9) = 2.40, SEM = 5.29, *p* = .04, *d* = 1.60 for the difference in Experiment 1a, *t* (13) = 2.16, SEM = 6.24, p = .05, *d* = 1.20 for the difference in Experiment 1b. Clearly then, Experiment 1b establishes that the load effects on TUT persist in the absence of any feedback and so cannot be attributed to the greater likelihood of error feedback in the high (compared to low) load condition.

A mixed model ANOVA with the between-subject factors of experiment (Experiments 1a and 1b) and the within-subject factor of load (low, high) revealed that the somewhat surprising numerical trend for an increase (rather than decrease) in the rate of TUT reports in Experiment 1a (*M* = 55.25%) compared with Experiment 1b (*M* = 50.75%) was not significant and neither was the interaction of experiment and load (both *Fs* < 1). The finding that TUT reports were clearly not reduced with the presence of feedback (in Experiment 1a) does not rule out the possibility that feedback can reduce TUT reports when subjects are explicitly instructed to use the feedback as a reminder to stay on task (cf. Manly et al., 2004). However, by demonstrating clear effects of load on TUT in paradigms both with and without feedback we have ruled out the any alternative interpretation of these load effects as being driven by the greater levels of feedback in the high load condition.

In addition, we note that, as each trial was triggered by the response to the previous trial, the greater RTs in the high load condition would have also resulted in a slower stimulus presentation rate in this condition. However, as previous studies (e.g., Antrobus, 1968; Giambra, 1995) have consistently reported increases in TUT as stimulus presentation rate decreases, our finding of a decrease in TUT reports under high load cannot be accounted for by the slower stimulus presentation rate in this condition.

Thus, Experiment 1 establishes that perceptual load, already known to be a powerful determinant of the processing of task-unrelated stimuli in the external world, may also determine the level of internal distractors in the form of task-unrelated thoughts. As no error feedback was given in Experiment 1b, these effects cannot be attributed to any effect of feedback (e.g., in capturing attention towards the task or in increasing the overall level of motivation in the high load compared to the low load condition).

## 3 Experiment 2

In Experiment 2 we sought to rule out any component of verbal working memory load in our task. As we used letters as search stimuli in Experiment 1 it could be argued that, in addition to increased perceptual demands, the verbal working memory demands in the high load condition (in which participants were required to discriminate the target letter from amongst five different non-target letters) were also greater than those in the low load condition (in which the five non-target letters were all Os and therefore only two letters could potentially be verbalized). The reduced level of TUTs under high load therefore could have simply reflected the suppression of verbal TUTs by participants’ internal verbalization of the larger letter search set under high load. To rule out this possibility, in Experiment 2 we used Hebrew letters for the search stimuli and recruited English participants who were unfamiliar with Hebrew, so that our search stimuli now appeared as meaningless shapes in both of the low and high load conditions.

### 3.1 Method

#### 3.1.1 Participants

Twelve new participants (two males, 8–23 years old) participated in Experiment 2. None of the subjects were able to read Hebrew or name any Hebrew letters.

#### 3.1.2 Stimuli and procedure

**Fig. 2:**
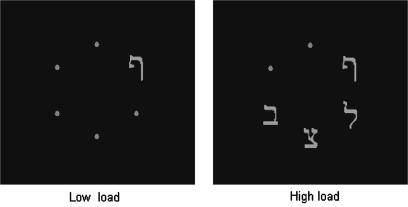
Example stimulus displays with low and high perceptual load in Experiment 2.

All stimuli and procedure were identical to those used in Experiment 1a, with the exception that Hebrew letters were used in place of the search letters of the previous experiments (see Fig. 2 for an example stimulus display). The target letters were ‘’ (mapped to the response key 2) and ‘’ (mapped to the response key 0). In the low load condition the non-targets were all small dots (subtending 0.2° × 0.2°). In the high load condition three Hebrew non-target letters, selected at random from the set , , ,  were presented with equal likelihood in three of the five positions not occupied by the target, with the remaining two positions occupied by small dots. All letters subtended between 0.6° and 0.9° vertically by 0.4° horizontally and were presented in gray for 100 ms exposure duration as before.

A set size of four was used in the high load condition as pilot testing had indicated that, using the Hebrew stimuli with non-Hebrew speaking participants, this set size was sufficient to induce an increase in RTs and error rate of a similar magnitude to that associated with the increase in load in Experiment 1. In addition, in order to ensure that the high load condition did not involve a greater verbal processing load than the low load condition (due to any verbal processing of the added non-target symbols) we asked three independent judges to complete the eight high load blocks used in the actual experiment (following the same experimental procedure) and, immediately following the final trial, to answer the following questions:

1. Did you, at any point during the experiment, assign verbal names to any of the symbols that you saw on screen? If so please could you write these down on the sheet of paper beside you, and also indicate whether these were target symbols or non-target symbols.
2. Did you, at any point in the experiment, have verbal thoughts relating to specific symbols? If so please could you write them down, indicating whether each thought was about a target or non-target symbol.

Although two out of the three judges indicated that they had assigned a verbal label to one of the two target symbols (“shoe” or “boot”), none of the judges had assigned any verbal names or reported any verbal thought associated with the non-target symbols. Note that any verbal demands associated with the target letters remained constant across the two load conditions.

### 3.2 Results

As in Experiment 1, the RT and error data showed the expected effects of perceptual load. RTs under high load were significantly longer (*M* = 719 ms) than under low load (*M* = 547 ms), *t* (11) = 7.87, SEM = 21.89, *p* < .001, *d* = 4.75 for the difference. The PE rate was also significantly higher under high load (*M* = 10.25%) than under low load (*M* = 6.41%), *t* (11) = 2.34, SEM = 1.64, *p* = .039, *d* = 1.41 for the difference.

The key finding of this experiment was that the participants again reported less TUTs in the high perceptual load condition (*M* = 53.15%), compared to low load condition (*M* = 65.62%), *t* (11) = 2.43, SEM = 5.10, *p* = .033, *d* = 1.47 for the difference (see Fig. 1). Thus, Experiment 2 establishes that the effect of perceptual load on the rate of TUT reports does not require the use of familiar verbal material that could serve to load verbal working memory and thus directly block the mental workspace for thought.

## 4 Experiment 3

In Experiment 3 we examined whether the reduction in the rate of TUTs reported under high perceptual load reflects an effect of load on deliberate TUTs or on unintentional TUTs. Previous perceptual load research suggests that the effects of perceptual load would not be confined just to deliberate TUTs: Increasing perceptual load has previously been found to eliminate interference even from potent task-irrelevant distractors that interfere with task performance in conditions of low perceptual load despite participants’ attempts to ignore them (e.g., Forster & Lavie, 2008a; Lavie, 1995) This leads us to predict that high perceptual load would be capable of reducing the rate of unintentional intrusions by TUTs. Alternatively, however, one might argue that for internal sources of irrelevant distraction high perceptual load could merely reduce the deliberate intention to engage in TUT (for example, because the high load task may be less boring) but would have no effect on those TUTs that intrude into the mind unintentionally. To examine this issue, in Experiment 3 participants were probed as to whether any task-unrelated thought they may have had was deliberate or unintentional.

### 4.1 Method

#### 4.1.1 Participants

Eighteen new participants (five males, 18–33 years old) participated in Experiment 3.

#### 4.1.2 Stimuli and procedure

The stimuli and procedure were the same as those used in Experiment 1a, with the exception that following the response to the thought probe the participants were presented with an additional probe asking them to indicate whether the reported TUT was deliberate or unintentional by pressing either the X or C keys respectively (or by pressing the A key if no TUT was reported). The following explanation of deliberate and accidental TUTs was given to the participants before they started the experiment: “Sometimes people may, for one reason or another, deliberately allow their mind to wander from a task that they are performing. This is a deliberate TUT. Other times a person may be really trying to concentrate on a task but somehow accidentally their mind wanders off to think about something else, unrelated to the task. This is an unintentional or accidental TUT.” Participants were informed that there was no right or wrong response to this question and that they should simply be honest.

### 4.2 Results

As in the previous experiments, RT and PE data showed the expected effects of perceptual load. RTs were longer in the high load condition (*M* = 679 ms) compared to the low load condition (*M* = 516 ms), *t* (17) = 14.07, SEM = 11.95, *p* < .001, *d* = 6.82 for the difference. PE rates were also significantly higher in the high load condition (*M* = 10.17%), compared to the low load condition (*M* = 4.61%), *t* (17) = 5.76, SEM = 0.97, *p* < .001, *d* = 2.79 for the difference.

**Fig. 3:**
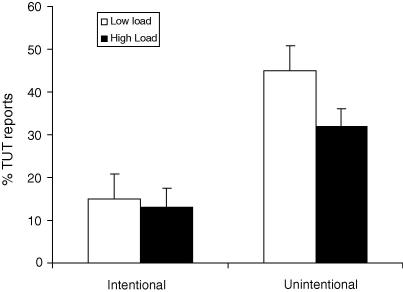
Percentage of probes at which intentional and unintentional task-unrelated thoughts (TUT) were reported under high and low perceptual load in Experiment 3.

The TUT reports were entered into a within-subject ANOVA with the factors of perceptual load (low, high) and intention (deliberate, unintentional). As can be seen in Fig. 3, the ANOVA showed a main effect of perceptual load that replicated the results of previous experiments: The rate of TUTs reported under low perceptual load (*M* = 60.06%) was significantly reduced under high perceptual load (*M* = 44.87%), *F* (17) = 13.05, MSE = 81.65, *p* = .002, $\eta_{p}^{2}$ = .356. There was also a main effect of intention: unintentional TUTs were reported on a significantly greater percentage of trials (*M* = 38.28%) than deliberate TUTs (*M* = 13.89%), *F* (17) = 9.41, MSE = 1083.95, *p* = .007, $\eta_{p}^{2}$ = .434. The interaction between load and intention was not significant, *F* (17) = 2.33, MSE = 222.20, *p* = .145, $\eta_{p}^{2}$ = .120. However, the low rate of deliberate TUTs found in this experiment makes this measure insensitive to show any effect or interaction. For this reason we confine our conclusion to the unintentional TUTs only. With respect to these, the results of Experiment 3 clearly show that high perceptual load can reduce intrusions by unintentional task-irrelevant thoughts. This finding has important implications both for predicting the ability to pay attention to a task with minimal distraction by unintentional TUTs in daily life and for clinical populations (e.g., ADHD). We discuss these in greater detail in Section 6.

## 5 Experiment 4

Having established the effects of perceptual load on mind-wandering, in Experiment 4 we sought to relate the effects of perceptual load on the processing of task-irrelevant and potentially distracting information from internal and external sources. We thus included external task-irrelevant distractors, adding flanker letters that could be incongruent or congruent with the target to the paradigm used in Experiments 1–3, and assessed the effects of perceptual load on both the internal (rates of TUT) and external (response-competition effects) forms of potential distraction.

### 5.1 Method

#### 5.1.1 Participants

Twenty new participants (six males, 19–30 years old) participated in Experiment 4.

#### 5.1.2 Stimuli and procedure

All stimuli and procedure were the same as in Experiment 1a, with the exception that the distractor letters X or N, each subtending 0.8° × 0.5°, were presented to the left or the right of the letter circle (1.4° from the nearest circle letter). All combinations of distractor position, distractor identity, target position and target identity and load were fully counterbalanced.

### 5.2 Results

Table 1 presents the results. A 2 × 2 within-subjects ANOVA of the mean RT with the factors of distractor congruency and load revealed main effects of load, F (1, 19) = 82.56, MSE = 10140.6, *p* < .001, $\eta_{p}^{2}$ = .813, and distractor congruency, *F* (1, 19) = 16.11, MSE = 539.30, *p* = .000, $\eta_{p}^{2}$ = .449. Of most importance was the interaction between load and distractor congruency, *F* (1, 19) = 17.655, MSE = 319.6, *p* < .001, $\eta_{p}^{2}$ = .482, indicating that the distractor response-competition effect under low load (distractor effect *M* = 38 ms, *t* (19) = 5.13, SEM = 7.33, *p* < .001, *d* = 2.35) was significantly reduced with high load (distractor effect *M* = 4 ms, *t* < 1). These results replicate previous findings (e.g., Lavie & Cox, 1997), demonstrating a significant reduction in interference from external distractors under high perceptual load.

A 2 × 2 ANOVA on the PE rates also revealed main effects of load, *F* (1, 19) = 50.61, MSE = 46.73, *p* < .001, $\eta_{p}^{2}$ = .727, and distractor congruency, *F* (1, 19) = 32.90, MSE = 2.88, $\eta_{p}^{2}$ = .634, *p* < .001. Although the load × congruency interaction was not significant, *F* (1, 19) = 1.30, MSE = 8.09, *p* = .269, $\eta_{p}^{2}$ = .064, the numerical trends seen in Table 1 were in the same direction as the RT.

**Fig. 4:**
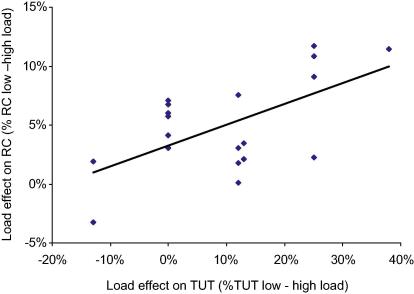
Correlation between load effects on internal distractors (TUT reports) and external (response competition) distractors. Response-competition (RC) effects were calculated as the percentage increase in RTs on incongruent trials compared to congruent trials.

As in Experiments 1–3, high perceptual load has also significantly reduced the rate of TUTs reported in this experiment (for the high load *M* = 41.25%, for low load *M* = 51.90%), *t* (19) = 3.43, SEM = 3.10, *p* = .003, *d* = 1.57 for the difference. Furthermore, there was a significant correlation between the effect of perceptual load on TUTs and on the distractor response-competition effects both when these were calculated as the mean *I*–*C* under each level of load, Pearson *r* (20) = .489, *p* = .029, and when individual differences in the baseline RT were accounted for by computing the percentage RT increase in the incongruent (vs. congruent) distractor conditions under the different levels of load, Pearson *r* (20) = .571, *p* = .009 (see Fig. 4). This correlation was not due to variations in the extent to which load has increased the demand of the search task as it remained significant when individual variations in the magnitude of the main effect of load on RTs were partialed out, *r* (17) = .491, *p* = .033 (for the same correlation with distractor response-competition effects calculated as percentages *r* = .599, *p* = .007).

The clear relationship between the magnitude of the load effects on internal (TUTs) and external (response competition) distractors across individuals suggests that these decreases in both internal and external distractors reflect one underlying focused attention ability driven by the level of perceptual load in the task.

Notice that the finding of a correlation between individual differences in the magnitude of the effect of perceptual load on internal and external distractors does not contradict previous load research by suggesting that perceptual load is less effective in reducing interference by external distractors for some individuals: Consistent with previous studies (e.g., Forster & Lavie, 2007), high perceptual load also eliminated distractor response-competition effects for all subjects in the present study (see Table 1), but the correlation indicates that perceptual load was most effective in reducing not only distractor effects but also TUTs for those individuals with the greatest levels of distractor effects in the conditions of low perceptual load.

## 6 General discussion

The present experiments establish that, like task-irrelevant external distractors, internal sources of distraction in the form of task-unrelated thoughts can also be modulated by the level of perceptual load in the task performed. This finding implies that, despite the obvious differences between TUTs and external distractors (e.g., TUTs are internally generated and may be drawn from long term memory processes, see Christoff, Ream, and Gabrieli (2004)), the process of the distraction of attention by task-irrelevant stimuli may involve common mechanisms (regardless of the nature of the source of distraction). The experiments further clarify that the reduction in the level of TUTs reported under high perceptual load is not simply due to a change in the demands on verbal working memory (Experiment 2), rate of responses and response feedback or the level of motivation (Experiment 1) or deliberate intention to engage in TUTs (Experiment 3).

As we have ruled out any alternative account for the effects of perceptual load in terms of a change in the extent to which the visual search task directly recruits components that are needed either for the actual production or the report of any thought (e.g., working memory, cf. Smallwood & Schooler, 2006; Teasdale et al., 1995), these results suggest the sharing of a central perceptual processing resource (attention) between task-unrelated thoughts and the high load search task in the manner predicted by the Load Theory. Notice that in this respect the effects of perceptual load demonstrated here are rather different to the effects of other task manipulations that have been previously found to reduce TUT reports. As we discuss earlier (in the General Introduction) the previous task manipulations shown to reduce TUT reports (dual task vs single task conditions, fast vs. slow stimulus presentation rates or conditions of high vs low working memory load) have directly drawn on either thought or response components. The reduction in TUT reports in those cases may therefore be due to direct interference with thoughts or the processes involved in reporting thoughts rather than to the enhanced focusing of attention on the task.

The correlation between the effects of perceptual load on TUTs and distractor response-competition effects (Experiment 4) further demonstrates the role of a common focused attention mechanism (driven by the level of perceptual load in the task) in determining the levels of both internal and external distractor interference. The present findings therefore demonstrate that the Load Theory of attention can be generalized to determine another potent form of distraction due to mind-wandering.

Our use of a probe-caught method that did not require the participants to continuously monitor for the occurrence of a task-unrelated thought, instead asking the subjects to report what they were thinking “just now”, makes it unlikely that the results could be attributed to alternative accounts in terms of load effects on either meta-awareness of thoughts (see Smallwood and Schooler (2006)) or on the encoding of thoughts into memory. As our self-report measure did require participants to be sufficiently conscious of their thoughts to be able to report them, however, it remains possible that the effects of load are restricted to conscious task-unrelated thoughts but that unconscious thought processes are unaffected by load.

Interestingly, a recent study demonstrates that perceptual load can modulate unconscious processing of external task-irrelevant stimuli. Bahrami et al. (2008) examined tilt after effects following adaptation to oriented gratings that were rendered invisible during adaptation with continuous flash suppression. Participants performed either a high or a low perceptual load task at fixation during adaptation to the invisible oriented gratings and showed reduced tilt after effects following the high compared to low load task, despite having no conscious perception of the gratings. It would therefore be an interesting avenue for future research to examine whether the effects of perceptual load on task-unrelated thoughts extend to unconscious thoughts.

As the first study of mind-wandering to directly manipulate task demands on attentional capacity, our conclusion that perceptual load plays a causal role in determining mind-wandering can both accommodate previous suggestions and guide future frameworks of mind-wandering. For example, it has been recently suggested that mind-wandering depends on the extent to which a task demands engagement with the external environment, and that the smaller rate of TUTs found in some studies using reading tasks compared to other studies that have used signal detection tasks is due to the greater level of task engagement in reading vs. signal detection tasks (Smallwood, Fishman et al., 2007).

Our present application of Load Theory to mind-wandering can clarify the concept of task engagement by suggesting that the level of task engagement would depend on the level of perceptual load in the task. Notice that this proposal does not rely on a taxonomy of tasks that are supposed to always demand greater or lesser levels of engagement. Instead we propose that any type of task may be prone to more or less TUT depending on the level of perceptual load it involves. For instance a signal detection task conducted under conditions of high perceptual load would be predicted to be less prone to mind-wandering than either the same task with low perceptual load or a reading task of a low perceptual load (e.g., repeated reading of the same words).

In this way, Load Theory offers a theoretical framework for determining levels of task-engagement and (conversely) task-unrelated thought. A fruitful direction for future research would be to explore this suggestion by measuring TUT whilst employing a previously established manipulation of perceptual load during a signal detection task (e.g., see Macdonald & Lavie, 2008).

By demonstrating the effect of perceptual load on task-unrelated thought, the present study represents a first stage in the application of load theory to internal distractors. Load theory makes a clear distinction between the effects of perceptual load and of executive control load on distractor processing, Whereas high perceptual load reduces distractor processing, high levels of cognitive load increase distraction due to reduced ability to suppress irrelevant information (e.g., see Lavie et al. (2004)). With respect to TUTs this would lead to an interesting counterintuitive prediction that high cognitive load would lead to an increased rate of TUT. A major obstacle, however, to testing this second prediction of load theory would be that, as has been discussed above, various components of executive control appear likely to be involved in the production of any thought (indeed it has been shown that increasing working memory load decreases TUT, Teasdale et al., 1993; Teasdale et al., 1995, presumably by directly interfering with the production of thought).

Finally, by identifying perceptual load as means by which to reduce levels of task-unrelated thoughts, the present findings also suggest that perceptual load may be useful in applied and clinical settings. As discussed above, task-unrelated thoughts have been shown to interfere with performance on a wide range of tasks – future research should investigate the possibility that perceptual load may protect against such interference by assessing the effect of TUTs on the performance of tasks modified to have high and low perceptual load. Another important implication of these results would be that higher levels of perceptual load could temporarily alleviate the unusually high levels of mind-wandering and task-unrelated thoughts associated with ADHD (Shaw & Giambra, 1993) or the intrusive task-unrelated thoughts associated with clinical disorders such as post-traumatic stress disorder (e.g., see Falsetti, Monnier, and Resnick (2005) and Obsessive Compulsive Disorder (e.g., see Clark and O’Conner (2005)). We are currently testing these hypotheses.

## Figures and Tables

**Table 1:** Experiment 4: Mean RTs (SE in parentheses) and percentage error rates as a function of distractor conditions and load, and mean percentage of task-unrelated thoughts (TUTs) as a function of load.

	Low load	High load
*I*		
RT (ms)	605 (29)	793 (36)
% Error	*7*	*17*

*C*		
RT (ms)	567 (24)	789 (36)
% Error	4	16
TUTs	41.25%	51.90%

*Note.I* = incongruent distractor, *C* = congruent distractor.

## Footnotes

1 Note that we restrict our review only to manipulations that produced significant effects on the level of TUT reported.

2 Note that the effects of perceptual load have previously been demonstrated using both manipulations of set size and manipulations of the complexity of processing demands (e.g., see Lavie (1995) for review see Lavie (2005)). We have chosen to use the search set size manipulation for this study as this manipulation has been shown not to interfere with working memory task performance, as discussed above.
